# 1179. PCV13 Pediatric Routine Schedule Completion and Adherence Before and During the COVID-19 Pandemic in the US

**DOI:** 10.1093/ofid/ofab466.1372

**Published:** 2021-12-04

**Authors:** Liping Huang, Jennifer L Nguyen, Johnna Perdrizet, Tamuno Alfred, Adriano Arguedas

**Affiliations:** 1 Pfizer, Inc., Collegeville, PA; 2 Pfizer Inc., New York, New York; 3 Pfizer Inc, Collegeville, Pennsylvania

## Abstract

**Background:**

Coronavirus Disease 2019 (COVID) mitigation measures may have unintended consequences, such as reduced or delayed access to routine immunizations. This study examined (1) PCV13 routine vaccination completion and adherence (C&A) among US infants before and during the COVID pandemic and (2) the relationship between primary dose C&A and booster dose C&A.

**Methods:**

Retrospective data from the Optum’s de-identified Clinformatics Data Mart Database were used to create 3 cohorts: C1, Pre-COVID; C2, During COVID; C3, Cross-COVID (Figure 1). The completion was defined as number of PCV13 doses received within 8 months of birth, and the adherence was defined number of doses received at ACIP recommended time (@2, 4, 6 months, +/- 5 days). Univariable logistic regression was used to compare the odds of primary dose C&A in cohorts C1 and C3 vs C2 and descriptive analyses were used to explore primary dose C&A in relation to booster dose C&A.

Figure 1: Study population and inclusion criteria

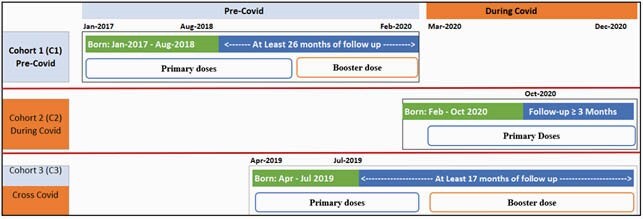

**Results:**

A total of 172,916, 70,049, and 34,854 infants were included in C1, C2, and C3. Among infants with > 8 months of follow-up from birth (N=132,183 for C1&C3, 16,522 for C3), 3-primary dose completion was statistically significantly higher before COVID than during COVID (crude OR = 1.10, 95% CI: 1.06-1.15). The 3-primary dose adherence was also higher before COVID than during COVID (crude OR = 1.10, 95% CI: 1.05-1.15). Among infants with ≥2, 4 and 6 months of follow-up, adherence of each individual dose was consistently higher before COVID than during COVID (1st dose: OR = 1.03, 95% CI: 1.01–1.04; 2nd dose: OR = 1.04, 95% CI: 1.01 – 1.06; 3rd dose: OR = 1.12, 95% CI: 1.08 – 1.15) (Table 1). Booster dose completion was higher in infants who completed or adhered to 3 primary doses than infants who completed or adhered to only 1 or 2 primary doses (Figure 2, Overall) and booster dose C&A was generally higher before COVID than during COVID (Figure 2, Cohort 1 vs. Cohort 3).

Table 1. Comparison of completion and adherence of primary dosing series per-COVID vs. during-COVID era

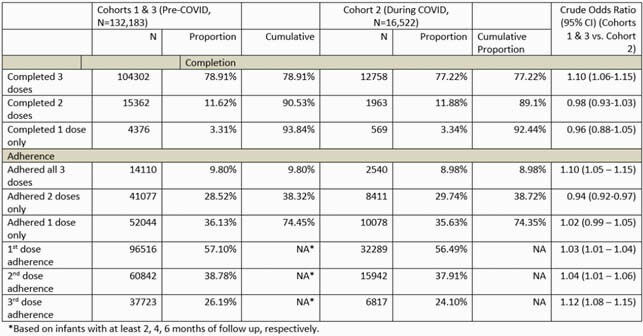

Figure 2: Booster dose completion and adherence in relation to primary dosing completion (A) and adherence (B)

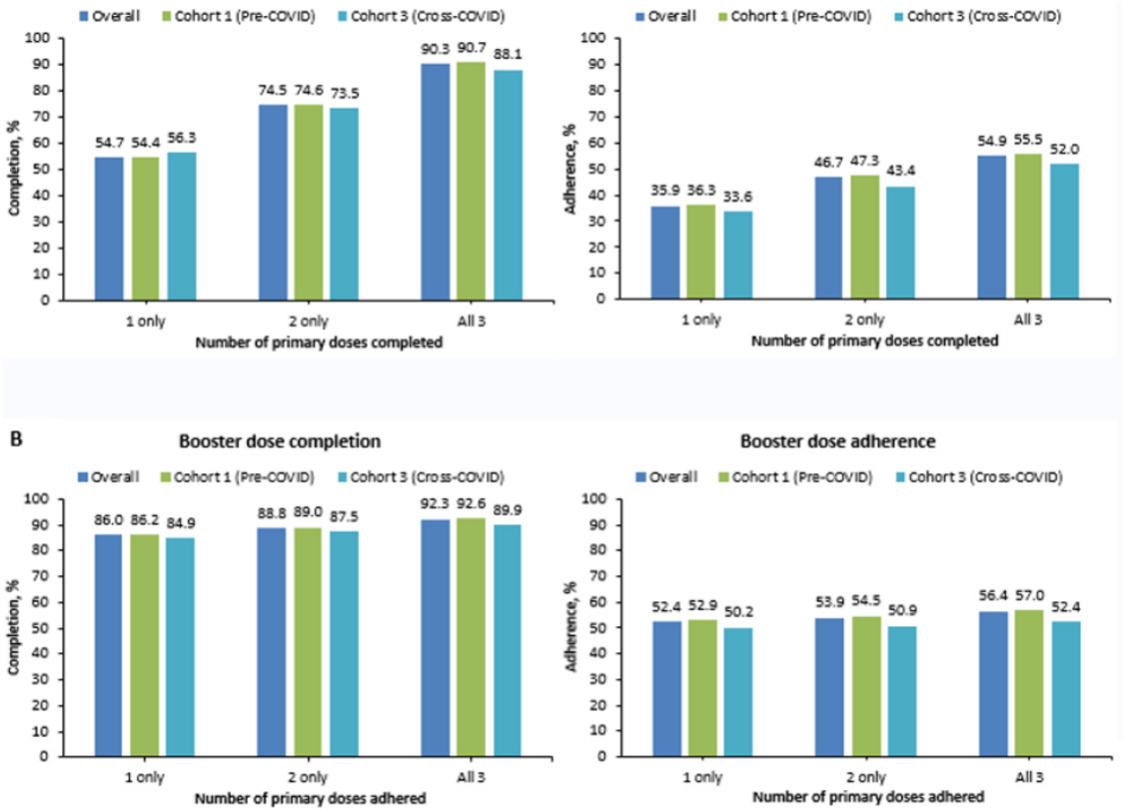

**Conclusion:**

These results indicated that PCV13 full completion was statistically lower during COVID, but the magnitude of the difference in infants was not extensive. Infants who completed or adhered to all three primary doses were more likely to complete or adhere to the booster dose. Further research is warranted as structured datasets mature to capture the full time span of COVID-19 mitigation measures.

**Disclosures:**

**Liping Huang, MD, MA, MS**, **Pfizer Inc** (Employee) **Jennifer L Nguyen, ScD, MPH**, **Pfizer Inc.** (Employee) **Johnna Perdrizet, MPH**, **Pfizer Inc** (Employee) **Tamuno Alfred, PhD**, **Pfizer Inc.** (Employee) **Adriano Arguedas, MD**, **Pfizer** (Employee)

